# Temporal analysis of Family Health Strategy indicators from the perspective of the Brazilian National Primary Health Care Policy

**DOI:** 10.1590/0102-311XEN042523

**Published:** 2023-10-09

**Authors:** Andressa Daiana Nascimento do Carmo, Silvia Lanziotti Azevedo da Silva, Estela Márcia Saraiva Campos

**Affiliations:** 1 Universidade Federal de Juiz de Fora, Juiz de Fora, Brasil.

**Keywords:** Family Health Strategy, Primary Care, Spatio-Temporal Analysis, Estratégia Saúde da Família, Atenção Básica, Análise Espaço-Temporal, Estrategia de Salud Familiar, Atención Básica, Análisis Espacio-Temporal

## Abstract

Throughout the three editions of the Brazilian National Primary Health Care Policy (PNAB), changes were made in relation to the structure of the Family Health Strategy (FHS), with emphasis on modifications concerning the priority nature of the FHS as an organization and care strategy in primary health care. The objective was to analyze temporal trends in indicators related to the FHS from the perspective of the three PNAB editions: 2006, 2011, and 2017. This is a descriptive study of the temporal trend of indicators selected from a logical model constructed by components related to the FHS in the three editions of the PNAB. The logical model was developed based on the components Territory/Enrollment, Teams, Work Process, Territory Planning and Management, and Care for Priority Groups by Family Health Teams, each one being represented by selected indicators. The construction of the national and regional time series between 2007 and 2020 was carried out using the Joinpoint software. Most of the indicators showed an upward trend in the first time segments identified by the models, followed by segments of stability or decrease, especially after the year 2017. The indicator Number of community health workers stands out, which decreased after 2017 in most geographical regions and in Brazil. The 2017 PNAB may have discouraged the continuation and expansion of the FHS as the priority model of primary health care, by allowing and financing new teamwork arrangements and processes.

## Abstract (pt)

Ao longo das três versões da Política Nacional de Atenção Básica (PNAB) alterações foram realizadas em relação à estruturação da Estratégia Saúde da Família (ESF), com destaque para modificações em relação ao caráter prioritário da ESF como estratégia de organização e de cuidado na atenção básica. O objetivo foi analisar tendências temporais de indicadores referentes à ESF sob o olhar das três versões da PNAB 2006, 2011 e 2017. Estudo descritivo de tendência temporal de indicadores selecionados a partir de modelo lógico construído por componentes referentes à ESF nas três versões da PNAB. O modelo lógico foi elaborado baseado nos componentes Território/Adscrição, Equipes, Processo de Trabalho, Planejamento e Gestão do Território, e Cuidados à Grupos Prioritários pelas Equipes de Saúde da Família, sendo cada um representado por indicadores selecionados. A construção das séries temporais entre 2007 e 2020, nacional e regionais, foi realizada utilizando o software Joinpoint. A maioria dos indicadores apresentou tendência de crescimento nos primeiros segmentos temporais identificados pelos modelos, seguidos por seguimentos de estabilidade ou queda, principalmente após o ano de 2017. Destaca-se o indicador Número de Agentes Comunitários de Saúde que apresentou queda após 2017 na maioria das regiões geográficas e no Brasil. A PNAB 2017 pode ter proporcionado um desestimulo à continuidade e ampliação da ESF como modelo prioritário da atenção básica, ao permitir e financiar novos arranjos e processos de trabalhos de equipes.

## Abstract (es)

A lo largo de las tres versiones de la Política Nacional de Atención Básica (PNAB) alteraciones fueron realizadas en relación con la estructuración de la Estrategia Salud de la Familia (ESF), con destaque para modificaciones en relación con el carácter prioritario de la ESF como estrategia de organización y de cuidado en la atención básica. El objetivo fue analizar tendencias temporales de los indicadores referentes a la ESF desde la perspectiva de las tres versiones de la PNAB, 2006, 2011 y 2017. Estudio descriptivo de la tendencia temporal de los indicadores seleccionados a partir del modelo lógico construido por componentes referentes a la ESF en las tres versiones de la PNAB. El modelo lógico fue elaborado basado en los componentes Territorio/Adscripción, Equipos, Proceso de Trabajo, Planificación y Gestión del Territorio y Atención a Grupos Prioritarios por Equipos de Salud de la Familia, siendo cada uno representado por indicadores seleccionados. La construcción de las series temporales entre 2007 y 2020, nacional y regional, se realizó utilizando el *software* Joinpoint. La mayoría de los indicadores presentaron una tendencia de crecimiento en los primeros segmentos temporales identificados por los modelos, seguidos por segmentos de estabilidad o la caída, principalmente después del año 2017. Se destaca el indicador Número de Agentes Comunitarios de Salud que presentó una caída después de 2017 en la mayoría de las regiones geográficas y en Brasil. La PNAB 2017 puede haber proporcionado un desaliento a la continuidad y ampliación de la ESF como modelo prioritario de la atención básica, al permitir y financiar nuevos arreglos y procesos de trabajo de los equipos.

## Introduction

The Family Health Program (FHP) was launched in Brazil in 1994 as a proposal for expanding access to healthcare services, based on the work of multidisciplinary teams responsible for an enrolled population ^1^. By June 2004, the FHP was already present in 84% of Brazilian municipalities ^2^. In 2019, the Brazilian Ministry of Health registered 45,798 family health teams (FHT) eligible to be financed by the Federal Government ^3^. And in 2006, with the publication of the first Brazilian National Primary Health Care Policy (PNAB), the FHP began to be considered a priority strategy for strengthening primary health care (PHC) in the country, and was renamed Family Health Strategy (FHS) ^4^. The provision of care ceased to be focused on the disease to prioritize care centered on the individual, family, and community, reordering the PHC model ^1^.

The first PNAB expects the FHS, as the PHC guidance care model, to achieve problem-solving care, based on the principles of universalization, accessibility, care coordination, bonding, continuity, integrality, accountability, humanization, equity, and social participation ^4^. In the years 2011 and 2017, PNAB was reviewed, which brought about changes. In 2011, the FHS was maintained as a priority model and replacement for traditional PHC, expanding its coverage and resolvability ^5^. In 2017, the most update version of PNAB ^6^, significant changes were incorporated, especially in relation to the FHS as a priority model and the encouragement of other team configurations, such as traditional PHC, without the mandatory presence of community health workers (CHW) ^7^. The Family Health Support Centers (NASF) were renamed Expanded Family Health Care and Primary Care (NASF-AB), and were now able to also support traditional PHC teams, not only the FHS, as initially recommended ^6^.

Another important aspect to consider is the scenario of political instability, especially as of 2016, which led to delays in federal transfers and spending cuts, culminating in the approval of the Constitutional Amendment (EC) that freezes spending on health and education, namely the *EC n. 95/2016* ^8^. Following the political moment, the *Previne Brasil* Program was approved in 2019, which changes the logic of financing PHC from the fixed and variable floors, per capita, and the municipal responsibility for the management of PHC, to a payment by registration of the enrolled population and good performance on indicators determined by the Brazilian Ministry of Health, which may impair the access of some population groups and aggravate the underfunding of PHC ^9^.

Several studies on PHC were carried out considering the FHS as its care model, with the objective of identifying its impacts on health indicators and learning more about the teams’ work process. A study conducted by Vieira ^10^ highlighted the importance of FHT in the eradication of leprosy and of the work process organized in multidisciplinary teams. Ferreira et al. ^11^ evaluated, in Belo Horizonte (Minas Gerais State), the knowledge of FHS professionals and support teams of health indicators, highlighting the importance of FHS in their good performance.

The positive nature of the FHS as a priority model for PHC is known, but there is still no information as to whether changes during the enactment of the three PNABs may have impacted its role. To analyze the behavior of the FHS from the perspective of the three PNABs becomes relevant for allowing the study on the nuances of each version of the policy and how they may have influenced the growth and consolidation of FHS as a care model for PHC over time. Thus, the objective of the present study was to analyze the temporal trend of FHS indicators from the perspective of the 2006, 2011, and 2017 PNABs, for Brazil and its regions.

## Methods

This is a descriptive time-series study on indicators related to the FHS, defined based on a logical model.

### Development of the Logical Model

To prepare the logical model (Figure 1), an analysis of the official 2006, 2011, and 2017 PNABs documents, available online, was carried out by three professional specialists. Two read the documents separately and listed important points from the FHS item in each PNAB. Afterwards, the specialists met at several times and, by consensus, identified five components that express the recommendation of the role of FHS as a preferred model: (I) Territory/Enrollment; (II) Teams; (III) Work Process; (IV) Territory Planning and Management; (V) Care for Priority Groups by FHT. For each component, the objectives and strategies adopted for its organization were identified, according to guidelines contained in each PNAB. Lastly, the third professional, who also read the documents focusing on the FHS, confirmed the identification of the five components, validating the model. Based on this logical model, it was possible to identify the framework of important components for configuring FHS as the care model of PHC, which could be measured by the selected indicators.

*** 2017:**
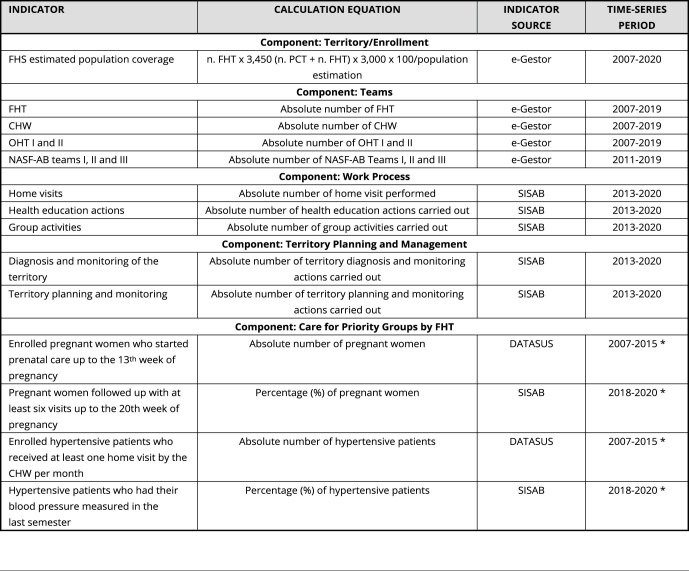
PNAB, item 3.3 - operation, (i): “*In addition to this population group, there may be other enrollment arrangements, depending on vulnerabilities, risks and community dynamics, allowing local managers, together with the teams that work in Primary Health Care and the Municipal Council or Local Health Council, the possibility of defining another population parameter under the responsibility of the team, which may be higher or lower than the recommended parameter, according to the specificities of the territory, ensuring the quality of care*” ^6^.

FHT: family health teams; NASF-AB: Expanded Family Health Care and Primary Care; OHT: oral health teams; PCT: primary care teams; PHC: primary health care; RAS: Healthcare Network.

### Selection and collection of indicators

To assess the components identified in the logical model of the PNAB editions, indicators that could reflect FHS as the priority model of PHC were selected. The selection of indicators was based on the Pact on Primary Care Indicators ^12^. Values of the indicators for each monitored year were collected from public databases of the PHC information systems (Primary Health Care Information and Management - e-Gestor, Information System for Primary Health Care - SISAB) and from the Tabnet tabulator, available from the website of the Brazilian Health Informatics Department (DATASUS; https://datasus.saude.gov.br/informacoes-de-saude-tabnet/), in the Health Care section (Primary Health Care - Family Health - from 1998 to 2015). Data collection covered the period between 2007 and 2020, considering the values for the month of August of each year, depending on the availability of data. Tabnet/DATASUS was replaced by the e-Gestor/SISAB databases due to the change in the data records and the financing model of the Brazilian PHC, replacing the Primary Health Care Information System (SIAB), and the indicators were adjusted to the change. Most of the indicators, even with the change in the information system, were collected from the same system, avoiding inconsistency in the records. Only the indicators of Care for Priority Groups by FHT were collected from different systems, but the information was not incorporated into the same timeline, preventing indicators with different calculation metrics from being analyzed together. The indicators related to the Teams component were only available from the e-Gestor system until 2019, and the monitoring until this year was then considered.

The Number of FHS Teams indicator was considered the total number of teams Accredited by the Brazilian Ministry of Health, available on SISAB, including teams with a workload of 40 hours per week. The 20-hour teams, considered primary care teams (PCT), were disregarded.

The selected indicators were collected for Brazil and for each of its geographical regions, North, Northeast, Central-West, Southeast, and South, and were tabulated in an Excel (https://products.office.com/) spreadsheet. Data for each of the selected indicators were interpreted according to the guidelines available from the Technical Notes of each information system.

As they are indicators available from public databases, the study did not require approval by the research ethics committee. The selected indicators, calculation indications, consulted databases, and collection period are described in Box 1.

**Box 1:** Indicators representing the components related to the Family Health Strategy (FHS) according to the logical model of analysis.

INDICATOR	CALCULATION EQUATION	INDICATOR SOURCE	TIME-SERIES PERIOD
Component: Territory/Enrollment			
FHS estimated population coverage	n. FHT x 3,450 (n. PCT + n. FHT) x 3,000 x 100/population estimation	e-Gestor	2007-2020
Component: Teams			
FHT	Absolute number of FHT	e-Gestor	2007-2019
CHW	Absolute number of CHW	e-Gestor	2007-2019
OHT I and II	Absolute number of OHT I and II	e-Gestor	2007-2019
NASF-AB teams I, II and III	Absolute number of NASF-AB Teams I, II and III	e-Gestor	2011-2019
Component: Work Process			
Home visits	Absolute number of home visit performed	SISAB	2013-2020
Health education actions	Absolute number of health education actions carried out	SISAB	2013-2020
Group activities	Absolute number of group activities carried out	SISAB	2013-2020
Component: Territory Planning and Management			
Diagnosis and monitoring of the territory	Absolute number of territory diagnosis and monitoring actions carried out	SISAB	2013-2020
Territory planning and monitoring	Absolute number of territory planning and monitoring actions carried out	SISAB	2013-2020
Component: Care for Priority Groups by FHT			
Enrolled pregnant women who started prenatal care up to the 13^th^ week of pregnancy	Absolute number of pregnant women	DATASUS	2007-2015 *
Pregnant women followed up with at least six visits up to the 20^th^ week of pregnancy	Percentage (%) of pregnant women	SISAB	2018-2020 *
Enrolled hypertensive patients who received at least one home visit by the CHW per month	Absolute number of hypertensive patients	DATASUS	2007-2015 *
Hypertensive patients who had their blood pressure measured in the last semester	Percentage (%) of hypertensive patients	SISAB	2018-2020 *

CHW: community health workers; DATASUS: Brazilian Health Informatics Department; FHT: family health teams; NASF-AB: Expanded Family Health Care and Primary Care; OHT: oral health teams; PCT: primary care teams; SISAB: Information System for Primary Health Care.

* Regarding the indicators of the component Care for Priority Groups by FHT no data for the years 2016 and 2017 were found in the consulted databases.

### Time-series analysis

The calculation of the time series for each indicator was performed using the joinpoint regression model, using the Joinpoint time trend analysis software (version 4.9.0.0; https://surveillance.cancer.gov/joinpoint/). The joinpoints established by the models connect several different lines through the “join points”, indicating changes in the trend and identifying an increase or decrease in the values of the indicators over the period evaluated for each one.

The assessment of the trends was based on the values of the annual percent change (APC), which reflects the change in the indicators in the analysis segments defined by the software, and the average annual percent change (AAPC), which reflects the average of the trend of the monitoring period according to the availability of the years of records of the indicators in the information databases. Positive APC and AAPC values indicate an upward trend; and negative values, a downward trend, when significant. The significance of the trends was verified by the 95% confidence interval (95%CI). When the APC and the AAPC were not significant, stability was considered.

## Results

Time series were constructed for 15 indicators, covering the five components of the logical model. All indicators were analyzed considering Brazil and its five regions, totaling 90 time series. When analyzing the time series, we identified different trends for each indicator and different trends in the same indicator in relation to Brazil and the geographical regions. Overall, the indicators showed an upward trend throughout the period, but this increase was greater in the initial years of the series and, in the following segments, we observed stability and even a decrease such as in relation to the number of CHWs.

Table 1 shows the trends, based on the APC and AAPC values, of the time series constructed with national data. We observed a total upward trend (AAPC) for the following indicators: FHS coverage, number of FHT, total number of oral health teams (OHT), total number of NASF-AB teams, number of actions aimed at diagnosing, monitoring, and planning the territory, and number of home visits. The other indicators showed stability in the AAPC analysis. Considering the time-series segments (APC), for most indicators, we observed an increase only at the beginning of the monitoring, and in the final years, trends of stability or decrease were identified.

**Table 1:** Temporal trends of the indicators selected by components of the logical model of analysis of the Brazilian National Primary Health Policy (PNAB), Brazil, 2007 to 2020.

Indicator	Segment	Initial year	Final year	APC	95%CI	APC trend	AAPC *	95%CI	AAPC trend
Component: Territory/Enrollment									
FHS population coverage (% of population covered by FHS)	1	2007	2018	3.08 **	2.5; 3.7	Upward	1.9 **	0.6; 3.2	Upward
	2	2018	2020	-4.42	-12.5; 4.4	Stability			
Component: Teams									
Number of CHW	1	2007	2016	2.6 **	1.5; 3.8	Upward	0.1	-1.5; 1.6	Stability
	2	2016	2019	-7.3 **	-13.0; -1.3	Downward			
Number of FHT	1	2007	2018	4.5 **	3.8; 5.2	Upward	3.1 **	1.7; 4.5	Upward
	2	2018	2019	-4.5	-13.3; 5.3	Stability			
Number of OHT I and II	1	2007	2015	7.2 **	5.4; 9.0	Upward	3.3 **	1.6; 5.0	Upward
	2	2015	2019	-4.0	-8.5; 0.7	Stability			
Number of NASF-AB teams I, II and III	1	2007	2015	7.2 **	5.4; 9.0	Upward	3.3 **	1.6; 5.0	Upward
	2	2015	2019	-4.0	-8.5; 0.7	Stability			
Component: Work Process									
Number of home visits	1	2013	2015	1428.7 **	1,071.1; 1,894.4	Upward	132.4 **	120.3; 145.2	Upward
	2	2015	2020	9.4 **	3.1; 16.1	Upward			
Number of health education actions	1	2013	2016	492.0	-3.1; 3,514.5	Stability	77.0	-5.2; 230.6	Stability
	2	2016	2020	-28.4	-77.2; 124.8	Stability			
Number of group activities	1	2013	2016	332.5	-19.8; 2,233.3	Stability	42.4	-20.4; 154.8	Stability
	2	2016	2020	-38.1	28.7; 79.8	Stability			
Component: Territory Planning and Management									
Number of actions for territory diagnosing and monitoring	1	2013	2015	2,684.5 **	530.9; 2,990.9	Upward	165.0 **	96.5; 257.5	Upward
	2	2015	2020	3.4	-25.8; 44.2	Stability			
Number of actions for territory monitoring and planning	1	2013	2015	2,012.0 **	253.9; 2,504.9	Upward	131.0 **	61.2; 231.2	Upward
	2	2015	2020	-4.7	-36.1; 42.2	Stability			
Component: Care for Priority Groups by FHT									
Number of enrolled pregnant women starting prenatal care up to the 13th week	1	2007	2009	-24.9	-48.0; 8.5	Stability	-7.8 **	-14.3; -0.8	Downward
	2	2009	2015	-1.3	-7.2; 5.1	Stability			
% of pregnant women followed up with at least six visits up to the 20th week	1	2018	2020	21.4	-70.7; 403.4	Stability	21.4	-70.7; -403.4	Stability
Number of enrolled hypertensive patients with at least one home visit/CHW/month	1	2007	2015	-8.8	0.2; -2.3	Stability	-8.8	0.2; -2.3	Stability
% of hypertensive patients with blood pressure measured in the last semester	1	2018	2020	29.1	-80.2; -740.7	Stability	29.1	-80.2; 740.7	Stability

95%CI: 95% confidence interval; AAPC: average annual percentage change; APC: annual percent change; CHW: community health workers; DATASUS: Brazilian Health Informatics Departments; FHS: Family Health Strategy; FHT: family health teams; NASF-AB: Expanded Family Health Care and Primary Care; OHT: oral health teams; PCT: primary care teams; SISAB: Information System for Primary Health Care.

Note: initial year = initial year of the segment; final year = final year of the segment;

* AAPC is calculated for the entire monitoring period for each indicator, representing the total period expressed by the segments;

** Significant by 95%CI.

Some indicators showed insufficient records of data in the initial years of the monitoring, leading to the identification of an initial significant upward trend with the regularity for feeding the information system. This situation was observed in the indicators of the Work Process component: number of home visits, number of health education actions, and number of group activities; and of the Territory Planning and Management component, measured by the indicator number of actions aimed at diagnosing and monitoring the territory.

Regarding the indicators of the component Care for Priority Groups by FHT: monitoring of pregnant women and hypertensive patients, the change in the calculation of the indicators in the consulted information systems influenced the temporal trend. The number of enrolled pregnant women who started prenatal care up to the 13th week of pregnancy showed a downward trend, and the percentage of pregnant women followed up with at least six consultations performed up to the 20th week of pregnancy was stable. Conversely, the indicators related to the follow-up of hypertensive patients, in their different measurement methods, were stable. The lack of data from these indicators in the years 2016 and 2017 hindered the full assessment of the trend in the period selected for the study.

Table 2 shows the results of the trends by time segment and the entire period of analysis of the indicators by geographical regions that showed different behaviors from the national scenario. In the Central-West Region alone, the indicator population coverage by FHS showed an upward trend in the segments and throughout the period of the time series, being much higher in the first period (2007-2009), which also resulted in the upward trend in the number of FHT. There was a drop in the South Region considering the number of OHT as of 2017, unlike Brazil, which showed stability as of 2016. The Northeast Region had the lowest number of indicators with trends different from the national ones. Most of the selected indicators showed trends similar to the national ones in the segments and total monitoring, and their APC and AAPC values are presented in the Supplementary Material (https://cadernos.ensp.fiocruz.br/static//arquivo/suppl-e00042523-ing_3252.pdf).

**Table 2:** Differentiated time trends between geographical regions and Brazil, indicators of the logical model of analysis of the Brazilian National Primary Health Policy (PNAB), 2007 to 2020

Indicator	Segment	Initial year	Final year	APC	95%CI	APC trend	AAPC	95%CI	AAPC trend *
North									
Component: Teams									
Number of NASF-AB teams I, II and III	1	2011	2014	34.9 **	18.9; 53.1	Upward	16.8 **	12.0; 21.7	Upward
	2	2014	2019	7.1 **	1.2; 13.3	Upward			
Component: Work Process									
Number of health education actions	1	2013	2015	780.4 **	76.2; 4,299.0	Upward	74.3 **	26.0; 141.1	Upward
	2	2015	2020	-8.8	-36.6; 30.7	Stability			
Component: Territory Planning and Management									
Number of actions for territory monitoring and planning	1	2014	2017	93.8	-5.5; 297.2	Stability	29.2 **	2.5; 62.8	Upward
	2	2017	2020	-13.9	-58.0; 76.6	Stability			
Component: Care for Priority Groups by FHT									
Number of enrolled pregnant women starting prenatal care up to the 13th week	1	2007	2013	-8.2	-18.6; 3.5	Stability	-12.3	-23.8; 1.0	Stability
	2	2013	2015	-23.4	-62.4; 55.8	Stability			
Number of enrolled hypertensive patients with at least one home visit/CHW/month	1	2007	2015	-8.4 **	-13.2; -3.2	Downward	-8.4 **	-13.2; -3.2	Downward
% of hypertensive patients with blood pressure measured in the last semester	1	2018	2020	29.1 **	1.9; 63.6	Upward	29.1 **	1.9; 63.6	Upward
Northeast									
Component: Teams									
Number of NASF-AB Teams I, II and III	1	2011	2014	32.7 **	26.1; 39.8	Upward	14.0 **	12.0; 15.9	Upward
	2	2014	2019	4.0 **	1.6; 6.4	Upward			
Component: Care for Priority Groups by FHT									
Number of enrolled pregnant women starting prenatal care up to the 13th week	1	2007	2009	-36.1 **	-45.1; -25.7	Downward	-9.7 **	-12.4; -7.0	Downward
	2	2009	2015	1.3	-1.2; 3.9	Stability			
Central-West									
Component: Territory/Enrollment									
FHS Population Coverage (% of population covered by FHS)	1	2007	2009	10.0 **	4.2; 16.2	Upward	3.1 **	2.3; 3.9	Upward
	2	2009	2020	1.9 **	1.5; 2.2	Upward			
Component: Teams									
Number of CHW	1	2007	2016	2.2 **	0.6; 3.9	Upward	-0.7	-2.8; 1.5	Stability
	2	2016	2019	-8.8 **	-16.6; -0.4	Upward			
Number of FHT	1	2007	2009	13.3 **	3.7; 19.4	Upward	4.8 **	3.8; 5.9	Upward
	2	2009	2019	3.7 **	3.2; 4.2	Upward			
Component: Work Process									
Number of health education actions	1	2013	2015	1,496.6	-36.7; 4,189.7	Stability	131.8 **	20.9; 344.5	Upward
	2	2015	2020	7.2	-47.9; 120.6	Stability			
Number of group activities	1	2013	2018	134.8 **	46.3; 276.8	Upward	17.6	-23.2; 80.2	Stability
	2	2018	2020	-79.1	-97.5; 73.5	Stability			
Component: Territory Planning and Management									
Number of actions for territory monitoring and planning	1	2014	2018	73.5 **	23.7; 143.3	Upward	15.3	-4.9; 39.7	Stability
	2	2018	2020	-49.1	-82.5; 48.8	Stability			
Component: Care for Priority Groups by FHT									
Number of enrolled pregnant women starting prenatal care up to the 13th week	1	2007	2009	-37.8 **	-48.4; -25.1	Downward	-11.2 **	-14.4; -7.8	Downward
	2	2009	2015	0.1	-3.0; 3.3	Stability			
Southeast									
Component: Teams									
Number of CHW	1	2007	2017	3.5 **	3.0; 3.9	Upward	0.2	-0.6; 1.1	Stability
	2	2017	2019	-14.4 **	-19.9; -9.4	Downward			
Component: Work Process									
Number of health education actions	1	2013	2015	2,474.2 **	50.4; 3,963.1	Upward	129.8 **	29.6; 307.4	Upward
	2	2015	2020	-12.6	-53.7; 65.0	Stability			
Component: Care for Priority Groups by FHT									
Number of enrolled pregnant women starting prenatal care up to the 13th week	1	2007	2011	-10.6 **	-19.9; -1.2	Downward	-4.3	-9.0; 0.6	Stability
	2	2011	2015	2.4	-7.3; 13.1	Stability			
Number of enrolled hypertensive patients with at least one home visit/CHW/month	1	2007	2015	3.9 **	0.1; 7.5	Upward	3.9 **	0.1; 7.5	Upward
South									
Component: Teams									
Number of OHT I and II	1	2007	2016	6.3 **	4.8; 7.8	Upward	2.3 **	0.4; 4.2	Upward
	2	2016	2019	-8.9 **	-15.7; -1.6	Downward			
Component: Work Process									
Number of home visits	1	2013	2015	2,865.5 **	1,617.1; 5,733.2	Upward	343.6 **	295.4; 397.5	Upward
	2	2015	2020	-16.4 **	-26.4; 5.0	Downward			
Number of health education actions	1	2013	2016	416.4 **	89.0; 1,310.8	Upward	68.9 **	19.4; 139.0	Upward
	2	2016	2020	-26.9	-61.3; 38.0	Stability			
Number of group activities	1	2013	2016	480.9 **	13.6; 869.8	Upward	56.7	-10.8; 175.3	Stability
	2	2016	2020	-41.3	-79.1; 64.7	Stability			
Component: Care for Priority Groups by FHT									
Number of enrolled pregnant women starting prenatal care up to the 13th week	1	2007	2015	-5.5 **	-9.2; -1.5	Downward	-5.5 **	-9.2; -1.5	Downward
Number of enrolled hypertensive patients with at least one home visit/CHW/month	1	2007	2015	-5.7 **	-9.3; -0.9	Downward	-5.7 **	-9.3; -0.9	Downward

95%CI: 95% confidence interval; AAPC: average annual percentage change; APC: annual percent change; CHW: community health workers; DATASUS: Brazilian Health Informatics Departments; FHS: Family Health Strategy; FHT: family health teams; NASF-AB: Expanded Family Health Care and Primary Care; OHT: oral health teams; PCT: primary care teams; SISAB: Information System for Primary Health Care.

Note: initial year = initial year of the segment; final year = final year of the segment;

* AAPC is calculated for the entire monitoring period for each indicator, representing the total period expressed by the segments;

** Significant by 95%CI.

## Discussion

Based on the construction of a logical model related to family health by the analysis of the 2006, 2011, and 2017 PNABs, we identified important components related to the FHS as a priority care model of PHC. The behavior of each component was evaluated by the temporal trend of indicators, between 2007 and 2020. Overall, the indicators showed an upward trend in the early years, resulting from the driving potential of 2006 and 2011 PNABs, followed by recent stabilization or decline, especially after the last edition of the policy, in 2017.

The 2006 PNAB is considered to have been a milestone for the organization of PHC in the country, based on the FHS. Rarely has Brazil been able to define, at the national level, a policy that would drive comprehensive, accessible health care, capable of becoming widespread throughout the territory and provoking a reorientation of the care model proposed by the Brazilian Unified National Health System (SUS) ^13^. The 2006 PNAB represented great innovative potential by assuming the FHS as a strategy to strengthen PHC and organize the levels of care, establishing a multidisciplinary team that is the preferred gateway to the search for health care for populations. This team is responsible for coordinating care and organizing the Healthcare Networks (RAS), an orientation reinforced by the 2011 PNAB. However, the 2017 PNAB may have discouraged the continuation of the expansion of FHS as a priority model of PHC ^14^, as observed by the decrease or stability of several indicators analyzed at the end of the time series presented in this study.

When analyzed compared with other PNABs, the regression of the 2017 PNAB is clear, as it promotes relativization of universal coverage, segmentation of access, recomposition of teams valuing the traditional ones, weakening of the CHWs, reorganization of the work process, change in physicians’ working hours, non-mandatory specialization in family health, and the loss of the role of PHC as coordinator of the network care ^15^. The main effect of the 2017 PNAB on FHS, following the weakening of the presence of the CHWs, is the flexibilization of the organization and permissiveness of the traditional PHC model, prioritizing this model over FHS, favoring its dismantling and the detriment of the continuity, integrality, and coordination of care ^16^. The present study reinforces the reflection of these aspects in the trends of the indicators of the components of the logical model.

The first component of the logical model, Territory/Enrollment, showed, for Brazil and its regions, the upward trend of the FHS population coverage indicator. However, at the end of the historical series, coverage stopped growing and remained stable. These results are found in other studies. Coverage increased from 48% in 2007 to 64% in 2017 and, during a longer period of monitoring, from 4.4% in 1998 to 70% in 2017 ^17^. After 2017, the new PNAB, by allowing new PCT arrangements, with guaranteed financing, may have led to stagnation or even a decrease in the coverage of the FHS ^16^. In the city of Rio de Janeiro, FHS coverage decreased after 2017, from 62.6% to 40.5% in 2020 ^18^.

The second component identified was Teams represented by the indicators of the number of different teams operating in PHC. The number of FHT significantly increased between 1998 and 2017, which led to an increase in access to PHC in the country ^8^ ^,^ ^14^. However, the valorization of PCT at the expense of FHT in the 2017 PNAB is noteworthy, discouraging municipal managers from encouraging FHS, provoking its dismantling, and placing the FHS care model under threat ^14^ ^,^ ^16^. In Rio de Janeiro, the number of FHT that had grown since 2010 decreased from 1,180 FHT in 2017 to 789 FHT in 2020 ^18^. These results corroborate the upward trend found at the beginning of the time series in the present study, but which are stable at the end.

The Central-West Region showed no drop in the population coverage of the FHS and also in the number of FHT, which does not mean that it was not impacted by the 2017 PNAB. The region, especially the Federal District, showed significant growth in FHS coverage between 2006 and 2016 ^19^. In our study, the small increase observed in the time series in the final years was insufficient to constitute a decrease or stability, maintaining the growth pattern, even if it was lower.

Regarding the total number of OHT, there was an upward trend between 2007 and 2015, followed by stability until 2019. A study conducted by Melo et al. ^16^, analyzed the period from 2006 to 2011 and showed an increase in oral health coverage from 29.9% to 41.2%, in a period coinciding with the enactment of the first two PNABs. Oral health was included in a more egalitarian and decisive way after the 2011 PNAB, when the OHT was incorporated as part of the FHS ^20^. In Brazil, only 56.61% of the Brazilian population is covered by the OHT, and only 46.14% of the inhabitants are assisted by an OHT linked to the FHS ^20^. After the 2017 PNAB, OHT was no longer mandatorily integrated to the FHS, compromising the continuation of its expansion ^21^.

Another indicator of the Teams component is the number of CHWs, which showed an increasing trend in the initial years of the time series. In the State of Mato Grosso do Sul, the number of CHWs increased by 12% between the years 2008 and 2013, the period after the 2006 PNAB, which also includes the 2011 PNAB ^22^, corroborating the results of this study. However, the present study identified a downward trend in the number of CHWs as of 2018, as well as other studies that also identified a drop in the number of CHWs after the 2017 PNAB ^8^ ^,^ ^16^ ^,^ ^23^. This latest edition of the policy alters the performance profile of these professionals ^13^ ^,^ ^14^.

The number of NASF-AB teams is the last indicator referring to the Teams component, and showed an increase trend throughout the study period, which was higher at the beginning of the time series. The NASF-AB, created in 2008, was strengthened in the 2011 PNAB, receiving financial incentives, which increased access to professionals from these teams ^17^. The 2017 PNAB brings about important changes in the structure of the NASF-AB, which ceased to exclusively support FHS for all types of PCT, a form of diminishing the value of family health as a priority model ^17^.

The third component of the logical model is the Work Process, represented by indicators related to the actions taken to provide better care for the enrolled population: number of home visits, number of health education actions, and number of group activities. These indicators have the peculiarity that their records in PHC information systems were only initiated in the years 2013 and 2014, which, overall, generated a very significant upward trend at the beginning, followed by a smaller but still significant increase in subsequent segments.

The home visit is an activity that contributes to a greater and better relationship between the team and the enrolled population, focused on education and assistance, substantially contributing to the reduction of problems in the health-disease process ^6^. In the FHS, home visit constitutes an important health surveillance action, characterized by the development of actions for the promotion, prevention, and rehabilitation of individuals and families ^24^. In the city of Rio de Janeiro, home visit showed increasing numbers from 2010 to 2017, followed by a decrease, ranging from an annual average of 2.47 visits per 1,000 inhabitants in 2017 to 0.04 visits per 1,000 in 2020 20, as also observed in the North and South regions in the present study. The reduction in home visit points to the weakness of the FHS, considering that this strategy is capable of humanizing care and developing bonds between users, teams, and families, in addition to maintaining the FHS work in the logic of surveillance, promoting the early identification of diseases. Home visit is considered an action permeated by soft technologies such as communication, welcoming, bonding, dialogue, and listening ^25^.

The number of health education actions, another indicator of the Work Process, is considered a strategic action and is carried out in a planned manner, capable of strengthening the population’s education process, boosting the incorporation of health promotion and disease prevention attitudes, better adherence to treatment, activities aimed at popular participation, the monitoring and planning of health actions in the territory ^26^. Professionals belonging to the FHS carry out health education actions for the enrolled population at the most diverse times, such as blood pressure measurements, prenatal consultations, and immunization, seeking to promote self-care and the reduction of health conditions ^27^. This action should be encouraged in the FHT, avoiding decreases in its performance, especially by professionals who are not affiliated with the FHS.

The last indicator of the Work Process component is the number of group activities, and it is closely related to health education actions. A study conducted in the city of Recife (Pernambuco State) ^28^ showed the importance of health education groups in adhering patients to the treatment of chronic diseases and encouraging self-care, based on the expansion of spaces for the construction of shared knowledge ^28^.

In PHC, the planning of the actions that will be adopted in the territories of the family health units is one of the characteristics of the teams’ work in guiding decision-making to achieve the expected results. It is in this sense that, in the logical model, the component Territory Planning and Management included the indicators related to actions for diagnosing and monitoring the territory, and for planning the territory. Both showed similar trends, of growth in the initial years followed by stability. These indicators were also recorded late in the information systems consulted, which may explain the high growth trend in the first segments.

Team meetings are important for sharing knowledge and experiences, especially between CHWs and the other professionals that compose the FHT, providing knowledge and improvement of the epidemiological situation in the territory ^29^. They enable a more global and collective view of the cases identified in the enrolled population, providing integration, listening, professional appreciation, case planning, preparation of therapeutic projects, knowledge exchange, and consensus achievement ^25^. Conversely, such meetings can be perceived only as a bureaucratic tool and not as an effective instrument for developing the daily healthcare work ^30^. After a while, the bureaucratization of work may be responsible for reducing the growth of actions, which become less stimulating in the professional’s routine and are no longer carried out, giving way to assistance actions ^30^.

The last component of the logical model was Care for Priority Groups by FHT, analyzed by the indicators related to the monitoring of pregnant women and hypertensive patients. The indicators were maintained in the analysis even with changes in their calculation methodology, seeking a longer monitoring time. Most of the regional temporal trends for both indicators were downward or stable. These trends must be carefully analyzed, as these indicators were expected to show an upward trend, demonstrating the effectiveness of FHS actions.

Most pregnant women have more than six prenatal visits, as recommended by the Brazilian Ministry of Health. However, despite actions to prevent health problems during pregnancy, the number of complications during pregnancy, childbirth, and postpartum period are still alarming ^31^. Considering 13 Brazilian capitals, between 2007 and 2017, over 60% of pregnant women had six or more consultations at the PHC, with the vast majority concentrated in the South and Southeast regions of the country. Lower rates of prenatal visits were found in the North and Northeast regions ^32^. In the present study, the Northeast Region also showed a decrease in this indicator, which represents the most recent years of monitoring, according to the SISAB collection methodology. In the city of Rio de Janeiro, the average number of live births of women with seven or more prenatal visits showed a small reduction after the 2017 PNAB, from 80.2% of live births with seven or more prenatal visits, in 2017, to 77.8%, in 2020 ^18^. The annual average number of prenatal visits in PHC sharply decreased, from 6.1 consultations per live birth in 2017 to 0.2 consultations per live birth in 2020 ^18^. These results corroborate the stability and downward trends found in the present study.

Regarding the monitoring of hypertensive patients, Maciel et al. ^33^, in a study conducted in the north of the State of Minas Gerais, demonstrated that hypertensive patients followed up by FHT and who had guidance about their clinical condition showed better adherence to treatment. Aurélio et al. ^34^, in a study conducted in Brasília (Central-West Region), analyzed the perception of hypertensive users about FHT and identified that 43.7% considered the access to primary healthcare services to be poor before the FHS and, with the monitoring of the FHS, 99% of patients reported that the provision of care had improved ^34^.

To use the indicators for monitoring hypertensive patients and pregnant women by FHS, it was necessary to use records computed in the PHC information systems in different ways to achieve a longer evaluation period. Until 2015, the data collected for the indicators were extracted from Tabnet/DATASUS. With the publication of *Ordinance n. 2.979* ^35^ of November 12, 2019, which establishes the Previne Brasil Program, there was a change in the way PHC was financed in Brazil, and, consequently, in the way data were recorded. This new funding model replaced the PHC fixed and variable floors, moving from financing based on the number of inhabitants and FHS teams existing in the municipalities to a financing system based on the capitation of people by PHC teams and performance evaluation ^36^. This change may have reflected and influenced the behavior of the indicators in general.

Another important item related to the behavior of the indicators and their stability or decrease in the final years of the time series was the reduction of health funding, reinforcing the progressive underfunding of the SUS, currently considered defunding, considering that the resources are insufficient to maintain services, from its structure to the workforce ^37^. Austerity compromises universality, as the reduction of resources reduces the supply of services and impairs access, a reality observed in the results of the present study in the decrease and stability of indicators related to the FHS as of 2017, the year following the *EC n. 95* ^8^. The *EC n. 95*, together with the 2017 PNAB, removes funding from the Federal Government’s priority agenda for professionals such as CHWs and the NASF-AB team, assigning their funding under the responsibility of municipalities, which do not always have their own resources to do so ^38^.

The neoliberal agenda that has been preponderating in the Brazilian Ministry of Health as of 2017 removed incentives for the growth of PHC, such as the Brazilian National Program To Improve Access and Quality in Primary Care (PMAQ-AB), which in its three editions (2013, 2015, and 2018) promoted the certification of PHC teams and incorporated resources into the PHC variable floor, being a mechanism for inducing new practices and increasing quality, which was able to expand PHC coverage in many municipalities in the country during its operating years ^15^.

This study has limitations related to the instabilities of public data from the platforms available for research, which led to the unavailability of some information during the analyzed periods, including the lack of data on some indicators. The indicators that were analyzed up to 2020 may have been affected by the new coronavirus (COVID-19) pandemic. However, the information is from August 2020, with a few months of the health emergency, and there were no sudden changes and declines in activities compared with previous years in the trends, in such a way that we chose to keep the information from 2020 in the analysis. The article evaluates indicators related to process (more operational) and not to the impact and effect of actions on the enrolled population. Thus, future studies are necessary to assess the effect of PNABs on the provision of health care, with repercussions on the behavior of populations and the impact on indicators such as infant mortality and hospitalizations due to ambulatory care sensitive conditions.

The strength of the study is that it is a pioneering study in analyzing the role of the FHS from the perspective of the three published PNABs based on a logical model and its indicators, using temporal trend analysis. The study also problematizes one of the biggest challenges faced by PHC in Brazil, which is to have comparable indicators in the form of a time series. It emphasizes the fragility of a standardization that generates changes in the measurement metrics and in information systems, initially SIAB, then SISAB and e-Gestor, which contribute to discontinuities. Furthermore, the data from the historical series evidently reflected the bias in the federative agenda for Brazilian primary health care after the takeover of a group with a strongly neoliberal government project, in 2016.

## Conclusion

We identified important changes in the organization of PHC in Brazil, especially the FHS, over the time in which the PNABs were published. After a long period of encouraging the FHS as a model for the structuring of PHC, with the publication of the 2006 and 2011 PNABs, the publication of the 2017 PNAB places the FHS in a situation of risk, starting to have a team “competing” with it, namely the PCT. Such changes may cause setbacks in the advances achieved with the implementation of FHS, which could be reflected in the downward or stable trends in the analyzed indicators. They can also directly impact the attributes of PHC, by reducing access, impairing coverage, weakening the capacity to coordinate care and integrality, not allowing the resolution of most of the population’s problems at the first level of care, with consequences for network care by burdening other centers of the RAS.
